# The Cambridge University Course

**Published:** 1911-09-02

**Authors:** 


### THE CAMBRIDGE UNIVERSITY COURSE.

The degrees of Bachelor of Medicine (M.B.) and Bachelor of Surgery (B.C.) at this University are only conferred after the entire course of study with examinations for both has been carried out. The B.C. is, therefore, in itself a complete and registrable qualification to practise, and is frequently so used. To obtain either of these degrees it is necessary to pass five years in the study of medicine after registration as a student, and to reside for three years at the University. The professional examinations are three in number. The first comprises physics, chemistry, and biology; like similar examinations elsewhere, it is divided into two parts, which may be passed separately. The second absorbs at least two years' work, and frequently more. In the third, or Final examination, there are two part: one consists of pathology, bacteriology, pharmacology, etc., and may be taken as soon as various courses of lectures and laboratory work in those subjects have been duly attended; the other includes medicine, surgery, and midwifery with gynæcology, and it may not be attempted until the completion of the scheme of medical study, which includes three years' attendance on the practice of a recognised hospital.

When the third examination has been completed the student may take the degree of B.C. at once, but for the M.B. he must prepare and submit a thesis on some subject in medicine, surgery, or midwifery, selected by himself.

For the degree of M.D. it is necessary to have been M.B. at least three years, and to submit a thesis on some subject in medicine (not in surgery), which is a definite research or contribution to the knowledge of its subject.

